# A study of correlation of the dietary index for gut microbiota with non-alcoholic fatty liver disease based on 2007–2018 National Health and Nutrition Examination Survey

**DOI:** 10.3389/fnut.2025.1573249

**Published:** 2025-04-10

**Authors:** Yinda Wang, Binzhong Zhang, Lianzhong Feng, Chenxi Cao, Xiaoliang Fei

**Affiliations:** ^1^Department of Gastrointestinal Surgery, The Second Affiliated Hospital of Jiaxing University, Jiaxing, Zhejiang, China; ^2^Department of Radiology, The Second Affiliated Hospital of Jiaxing University, Jiaxing, Zhejiang, China

**Keywords:** dietary index for gut microbiota, non-alcoholic fatty liver disease, cross-sectional study, National Health and Nutrition Examination Survey, gut microbiota, dietary

## Abstract

**Objective:**

To explore the correlation of dietary index for gut microbiota (DI-GM) with non-alcoholic fatty liver disease (NAFLD).

**Methods:**

Data of 6,711 participants were extracted from the National Health and Nutrition Examination Survey (NHANES) during 2007–2018. A weighted logistic regression analysis was employed for assessment of the correlation of DI-GM with NAFLD, and a restricted cubic spline (RCS) analysis was implemented to examine potential non-linear associations. Subgroup analyses were conducted to identify particularly susceptible groups. Additionally, the synergistic effects of different DI-GM components on NAFLD risk was assessed by weighted quantile sum (WQS) regression.

**Results:**

The DI-GM exhibited statistically significant correlation with NAFLD [OR (95%CI):0.91 (0.85, 0.98), *p* = 0.015]. The results of the RCS analysis indicated a linear correlation of DI-GM and NAFLD (*p* = 0.810 for non-linearity). Further stratified analyses indicated that the negative correlation of DI-GM with NAFLD were significant and consistent for all subgroups. The results of WQS regression revealed that soybean (27%), refined grains (17%), coffee (16%), and red meat (9%) had the highest contribution weights to NAFLD.

**Conclusion:**

As an important tool for assessment of the influences of diet on gut microbiota, DI-GM is negatively correlated with NAFLD risk factors. Soybean, refined grains, coffee, and red meat are key factors influencing NAFLD. The direct correlation of DI-GM with NAFLD shall be explored and the effectiveness of prevention and treatment of NAFLD shall be evaluated by improving DI-GM scores via dietary interventions.

## Introduction

As a liver disease correlated to metabolic disorders, non-alcoholic fatty liver disease (NAFLD) is characterized by abnormal fat accumulation in liver induced by factors other than alcohol consumption (1–3). NAFLD comprises non-alcoholic fatty liver (NAFL) and non-alcoholic steatohepatitis (NASH), and it may further progress to hepatocellular carcinoma (1, 4–6). The incidence of NAFLD is approximately 38% globally, with regional variations ranging from 25.10% in Western Europe to 44.37% in Latin America (7). The prevalence of NAFLD and NASH in key regions such as China and USA is expected to increase by up to 30–56% from 2016 to 2030 (8). NAFLD has been demonstrated to be correlated with various factors, including insulin resistance (9, 10), lipotoxicity (11), inflammatory response (12), genetic polymorphisms (13), epigenetics (14), adipokines (15), myokines (15), hepatokines (15, 16), bile acids (17, 18), and gut microbiota (19–22).

Gut microbiota (GM), also known as the gut flora, refers to the microbial community in human intestine, and it plays a significant role in digestion and metabolism (23–25). Gut microbiota coexists symbiotically with the human body as the “second genome” (22). Previous studies have shown that GM is a dominant factor influencing incidence and progression of NAFLD (19–22). Diet determines the composition and diversity of GM (26–28). Furthermore, dietary interventions that can alter gut microbiota have attracted great attention (28). Kase et al. (26) reported a review of 14 dietary components with different influences on gut microbiota. The Dietary Index for the Gut Microbiota (DI-GM) was proposed for assessment of dietary quality associated with gut microbiota (26). Contrary to other dietary indices such as healthy eating index-2015 (HEI-2015) and alternative healthy eating index-2010 (AHEI-2010), DI-GM was established on the basis of gut microbiota but not food groups. Moreover, DI-GM had a positive correlation with urinary levels of intestinal diols and lactones, both of which are markers of gut microbiota diversity, indicating that DI-GM is associated with the diversity of GM (26). Hence, DI-GM can be used to effectively evaluate the influences of dietary patterns on GM (26) and serve as a standardized tool for diet assessment. Recent studies have shown that high DI-GM indicates low risk of accelerated aging (29), and DI-GM is inversely related to the prevalence of depression (30). Furthermore, compelling evidences have revealed that gut microbiota generates a variety of bioactive substances that interact with the host liver cells through the portal vein, which may lead to inflammation and further liver damage (31). However, the specific correlation of DI-GM with NAFLD remains unclear.

In this study, the correlation of DI-GM and NAFLD was investigated on the basis of the data extracted from the National Health and Nutrition Examination Survey (NHANES), and the potential of DI-GM which has been widely used for diet assessment for GM in prevention and dietary treatment of NAFLD was explored.

## Materials and methods

### Target community

**Figure 1:**
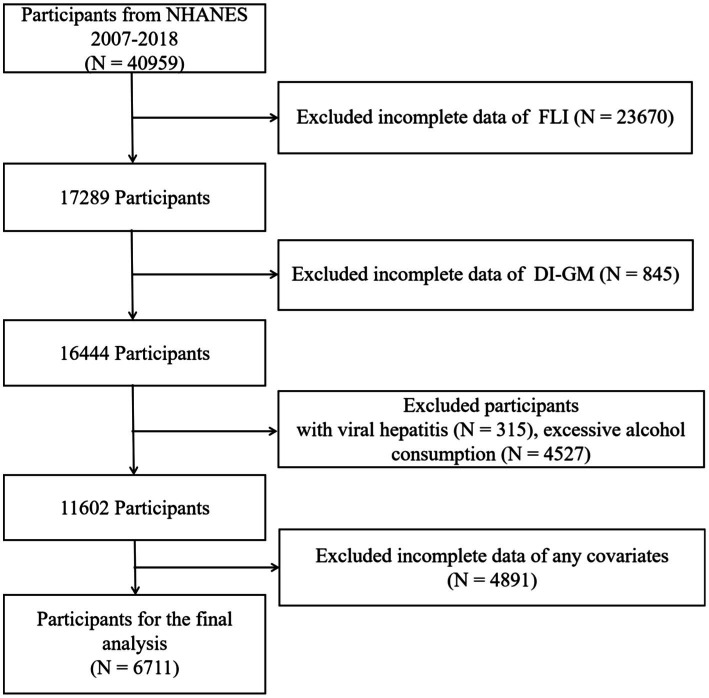
Flow diagram of study cohort selection.

Information acquired from public files for NHANES data cycles in 2007–2018 was analyzed. Across these cycles, 40,959 participants were enrolled, while the final cohort comprised 6,711 subjects only (Figure 1) as participants who provided incomplete data of fatty liver index (FLI) (*n* = 23,670), incomplete data of DI-GM (*n* = 845), with viral hepatitis (*n* = 315), excessive alcohol consumption (*n* = 4,527), or incomplete data for any covariate (*n* = 4,891) were excluded.

### Diagnosis of NAFLD

The FLI was employed for non-invasive diagnosis of NAFLD (32), and its accuracy and clinical significance in screening and diagnosis of NAFLD have been demonstrated in various studies (33). The FLI can be determined by: FLI = (e^0.953 × loge [Triglycerides (TG)] + 0.139 × Body mass index (BMI) + 0.718 × loge [*γ*-glutamyltransferase (GGT)] + 0.053 × Waist circumference (WC) – 15.745^)/(1 + e^0.953 × loge [TG] + 0.139 × BMI + 0.718 × loge [GGT] + 0.053 × WC – 15.745^) × 100, and the FLI of 60 or higher indicates a high risk of NAFLD (32).

### Assessment of DI-GM

Fourteen food or nutrients constituting key elements of DI-GM were identified (26). Beneficial elements include chickpeas, avocados, coffee, broccoli, fermented dairy products, cranberries, soybean, green tea, fiber, and whole grains. Meanwhile, detrimental elements such as refined grains, processed meat, red meat, and a high-fat diet were identified (26). The DI-GM was determined on the basis of the dietary recall data of NHANES 2007–2018, as shown in Supplementary Table S2. For food items with positive impacts on gut microbiota, 1 point was assigned when the gender-specific median was reached for intake, otherwise 0; for food items with negative impacts on gut microbiota, 0 was assigned when the gender-specific median was reached for intake or it accounts for 40% of total caloric intake (for high-fat diets), otherwise 1 point (26). A DI-GM score ranging from 0 to 13 (ranging from 0 to 9 for promoting the gut microbiota and 0 to 4 for negatively affecting the gut microbiota) was determined based on these scores, and then the participants were classified into Groups A (0–3), B (4), C (5) and D (≥6) (30), as presented in Supplementary Table S1.

### Covariables

In this survey, several potential confounding factors were considered: (1) Demographic factors: age, gender (male/female), race, marital status, and education. (2) Lifestyle factors: Smoking history (never, now, former). (3) Financial status: family poverty income ratio (PIR) (low, middle, high). (4) Health comorbidities: hypertension, cardiovascular disease (CVD), and diabetes mellitus (DM). (5) BMI (kg/m^2^). (6) Liver function indicators: Alanine aminotransferase (ALT) and aspartate aminotransferase (AST) (both in U/L). Detailed information could be achieved in Supplementary Table S2.

### Statistical analysis

Recommended sample weights were employed to enhance accuracy and undesired influences were mitigated by intricate multistage sampling. The results of categorical variables were denoted as weighted percentages, while the results of continuous variables were presented as weighted means ± standard deviations. To clarify the correlation of DI-GM and NAFLD, weighted multivariate logistic regression analyses were conducted. Herein, various confounding factors were considered and adjusted for. Three models were employed in this study. Model 1 was non-adjusted; Model 2 incorporated adjustments for key demographic variables; Model 3 additionally adjusted for BMI, PIR, smoking history, hypertension, DM, CVD, ALT and AST. Weighted restricted cubic spline (RCS) curves were introduced to Model 3 to investigate the correlation of DI-GM and NAFLD. Additionally, subgroup analyses were executed based on age, race, gender, education, marital status, smoking history, and common chronic diseases (hypertension and CVD). Considered the potential influences of physical activity, alcohol intake, medication use on the results. Accordingly, we further adjusted for these variables as outlined in Model 3 to assess the robustness of our findings in sensitivity analysis. Weighted quantile sum (WQS) regression models were employed for assessment of the synergistic effects of different DI-GM components on the risk of NAFLD, and the WQS index was determined on the basis of 60% training dataset, 40% validation dataset and 1,000 bootstrapping (34). To address the multicollinearity, weights were assigned to components according to their contributions to the results. The integrity of the statistical computations was validated using R software, with *p* < 0.05 denoting statistical significance.

## Results

### Characteristics of the participants

Table 1 shows the characteristics of the 6,711 subjects enrolled grouped by DI-GM. The average age was 51.06 ± 0.30 years. 51.28% were male and 48.72% were female. Significant differences were detected (*p* < 0.05) across DI-GM for demographic characteristics, financial status (PIR), lifestyle (smoking history), physical well-being (NAFLD, hypertension, CVD, DM), and anthropometric measures (BMI).

**Table 1:** Weighted characteristics of the study population according to the DI-GM group^a^.

Characteristics	DI-GM					*p*-value
	Overall	0–3	4	5	>=6	
Number	6,711	1,246	1,436	1,526	2,503	
Age (years)	51.06 (0.30)	49.76 (0.62)	50.21 (0.67)	49.88 (0.62)	52.72 (0.44)	<0.001
Sex (%)						0.205
Male	3,354 (51.28)	667 (54.11)	707 (51.71)	760 (52.36)	1,220 (49.23)	
Female	3,357 (48.72)	579 (45.89)	729 (48.29)	766 (47.64)	1,283 (50.77)	
Race (%)						<0.001
Non-Hispanic White	3,068 (70.32)	505 (65.29)	617 (65.49)	681 (69.75)	1,265 (75.26)	
Non-Hispanic Black	1,270 (10.25)	321 (15.02)	302 (12.35)	287 (10.31)	360 (7.12)	
Mexican American	893 (6.50)	179 (7.86)	224 (8.23)	225 (7.39)	265 (4.52)	
Other Race	1,480 (12.92)	241 (11.82)	293 (13.93)	333 (12.55)	613 (13.10)	
Educational attainment (%)						<0.001
High school or less	2,956 (36.26)	673 (47.22)	732 (43.09)	701 (37.27)	850 (27.51)	
More than high school	3,755 (63.74)	573 (52.78)	704 (56.91)	825 (62.73)	1,653 (72.49)	
Marital status (%)						0.030
Married or living with partner	4,282 (68.21)	794 (66.14)	865 (65.63)	958 (67.09)	1,665 (71.05)	
Living alone	2,429 (31.79)	452 (33.86)	571 (34.37)	568 (32.91)	838 (28.95)	
PIR (%)						<0.001
Low	1,270 (12.35)	266 (15.32)	311 (13.81)	333 (14.88)	360 (8.88)	
Middle	3,542 (48.21)	731 (55.54)	803 (52.66)	775 (44.44)	1,233 (44.98)	
High	1899 (39.43)	249 (29.14)	322 (33.52)	418 (40.68)	910 (46.14)	
Smoking status (%)						0.003
Never	4,143 (62.93)	730 (62.03)	860 (61.27)	954 (62.94)	1,599 (64.15)	
Now	871 (11.70)	188 (13.59)	215 (13.87)	216 (13.19)	252 (8.93)	
Former	1,697 (25.38)	328 (24.38)	361 (24.86)	356 (23.88)	652 (26.92)	
NAFLD (%)						<0.001
No	3,769 (56.27)	624 (49.59)	771 (52.70)	847 (53.28)	1,527 (62.67)	
Yes	2,942 (43.73)	622 (50.41)	665 (47.30)	679 (46.72)	976 (37.33)	
Hypertension (%)						0.021
No	3,558 (58.35)	623 (56.28)	740 (54.42)	824 (59.22)	1,371 (60.74)	
Yes	3,153 (41.65)	623 (43.72)	696 (45.58)	702 (40.78)	1,132 (39.26)	
CVD (%)						0.251
No	5,833 (89.09)	1,056 (87.90)	1,236 (87.86)	1,332 (89.55)	2,209 (89.97)	
Yes	878 (10.91)	190 (12.10)	200 (12.14)	194 (10.45)	294 (10.03)	
DM (%)						<0.001
No	5,075 (81.40)	886 (77.58)	1,048 (79.29)	1,173 (81.31)	1968 (84.15)	
Yes	1,636 (18.60)	360 (22.42)	388 (20.71)	353 (18.69)	535 (15.85)	
BMI (kg/m^2^)	29.11 (0.13)	29.74 (0.24)	29.79 (0.23)	29.43 (0.23)	28.32 (0.19)	<0.001
ALT (U/L)	24.01 (0.18)	24.11 (0.51)	24.65 (0.46)	23.80 (0.50)	23.76 (0.32)	0.436
AST (U/L)	24.19 (0.20)	23.83 (0.54)	24.37 (0.34)	23.75 (0.43)	24.50 (0.34)	0.509

DI-GM, dietary index for gut microbiota; PIR, family poverty income ratio; NAFLD, nonalcoholic fatty liver disease; CVD, cardiovascular disease; DM, diabetes mellitus; BMI, body mass index; ALT, alanine aminotransferase; AST, aspartate aminotransferase.

^a^Values are weighted means (standardized errors) or number of participants (weighted percentages) unless otherwise indicated.

### Association of DI-GM and NAFLD

Logistic modeling was executed to explore the association of DI-GM and NAFLD. According to Table 2, a significant negative correlation was identified [OR (95%CI): 0.88 (0.85, 0.92), *p* < 0.001], and the negative correlation remained robust after variable adjustment [OR (95%CI):0.91 (0.85, 0.98), *p* = 0.015] in Model 3, suggesting a 9% relative risk reduction per DI-GM unit which is modest at the individual level and meaningful at the population level. Furthermore, slight increase in DI-GM could also offer significant benefits for NAFLD, especially for the implementation of large scale and high-risk populations strategies. Meanwhile, the percentage of participants with DI-GM ≥ 6 had a significantly negative correlation with NAFLD [OR (95%CI):0.59 (0.48, 0.71), *p* < 0.001], and the negative correlation remained robust after variable adjustment [OR (95%CI):0.65 (0.47, 0.90), *p* = 0.010] in Model 3.

**Table 2:** Weighted multivariate logistic regression analysis of DI-GM and NAFLD^a^.

Characteristic	Model 1 OR (95%CI), *p*-value	Model 2 OR (95%CI), *P*-value	Model 3 OR (95%CI), *P*-value
DI-GM (continuous)	0.88 (0.85,0.92), <0.001	0.88 (0.85,0.92), <0.001	0.91 (0.85,0.98), 0.015
DI-GM (categorical)			
0–3	Reference	Reference	Reference
4	0.88 (0.73,1.07), 0.196	0.89 (0.74,1.08), 0.253	0.86 (0.62,1.20), 0.372
5	0.86 (0.70,1.05), 0.148	0.88 (0.72,1.08), 0.216	0.88 (0.64,1.22), 0.453
>=6	0.59 (0.48,0.71), <0.001	0.59 (0.48,0.72), <0.001	0.65 (0.47,0.90), 0.010
*P* for trend	<0.001	<0.001	0.013

DI-GM, dietary index for gut microbiota; PIR, family poverty income ratio; NAFLD, nonalcoholic fatty liver disease; CVD, cardiovascular disease; DM, diabetes mellitus; BMI, body mass index; ALT, alanine aminotransferase; AST, aspartate aminotransferase.

^aModel 1: unadjusted; Model 2: adjusted for age, sex, race, educational attainment, and marital status; Model 3: adjusted for age, sex, race, education attainment, marital status, BMI, PIR, smoking status, hypertension, DM, CVD, ALT and AST.^

### Non-linear correlation

**Figure 2:**
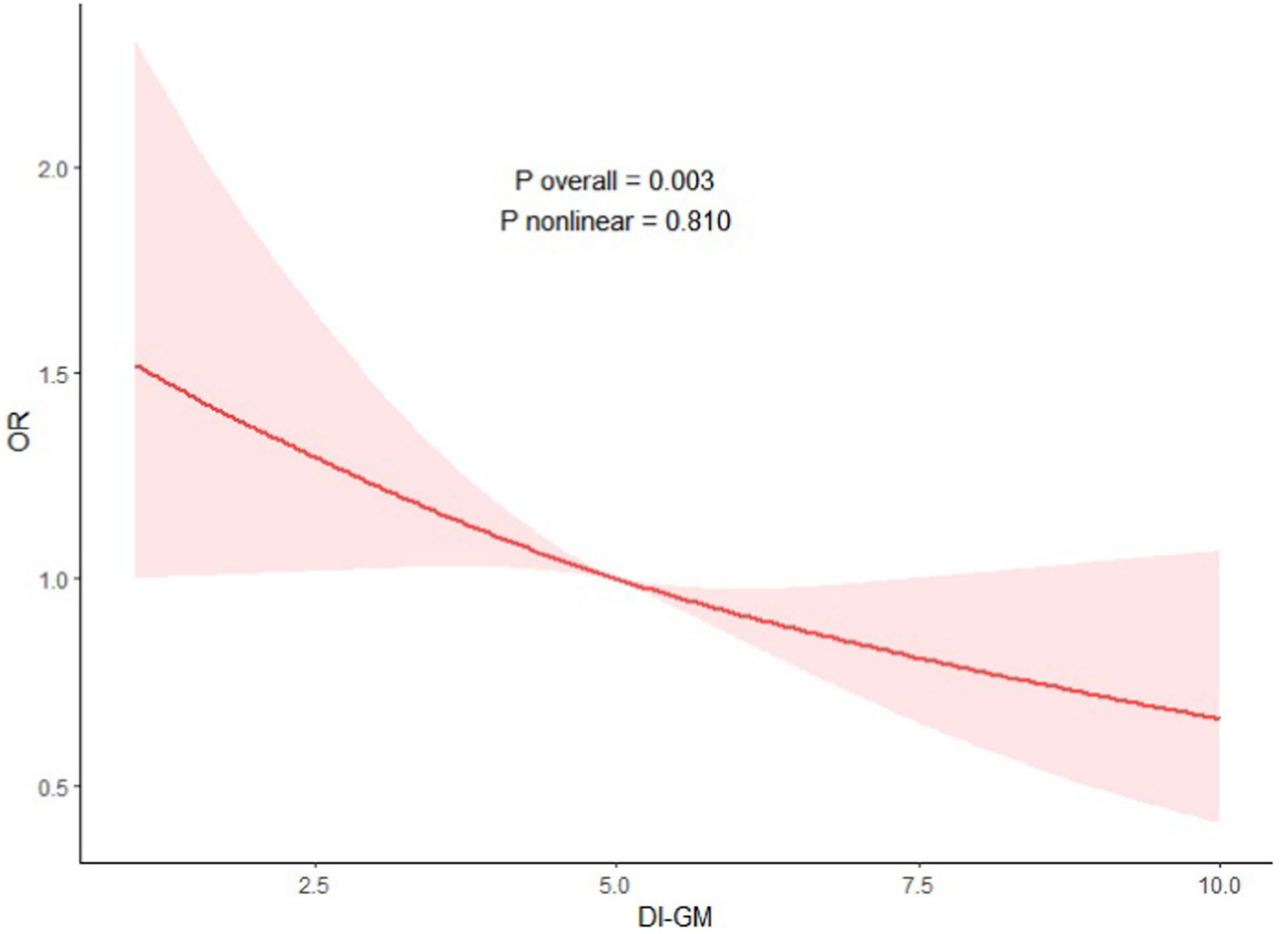
Association between DI-GM with NAFLD. Adjustment factors included age, sex, race, education attainment, marital status, BMI, PIR, smoking status, hypertension, DM, CVD, ALT and AST.

To explore the non-linearity of the associations of DI-GM and the NAFLD, a weighted multivariable–adjusted RCS analysis was executed in this study. As shown in Figure 2, no non-linear correlation of DI-GM and NAFLD was detected (*p* = 0.810 for non-linearity), and a negative dose–response correlation of DI-GM and NAFLD was identified (*p* = 0.003).

### Subgroup analyses and sensitivity analysis

**Figure 3:**
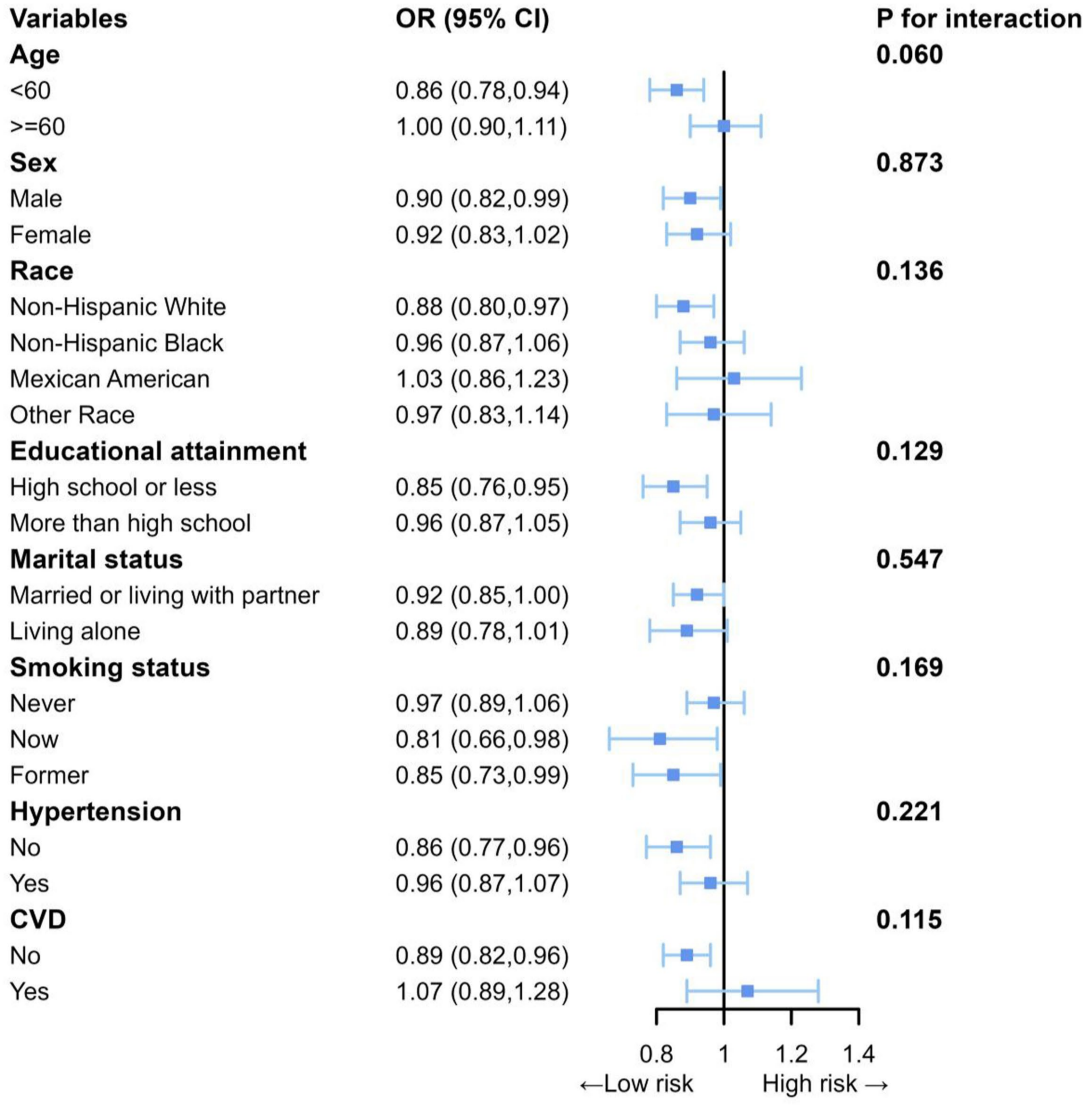
Subgroup analyses between DI-GM with NAFLD. Each subgroup analysis adjusted for age, sex, race, education attainment, marital status, BMI, PIR, smoking status, hypertension, DM, CVD, ALT and AST.

In this study, subgroup analysis was used to explore the specific correlations of DI-GM and NAFLD in different subgroups, indicating that the negative correlation of DI-GM and NAFLD was consistent (Figure 3). The results of the sensitivity analyses indicate that, even after further adjusting for physical activity, alcohol intake, medication use, the positive correlation between DI-GM and NAFLD persists (Supplementary Table S3).

### WQS regression

**Figure 4:**
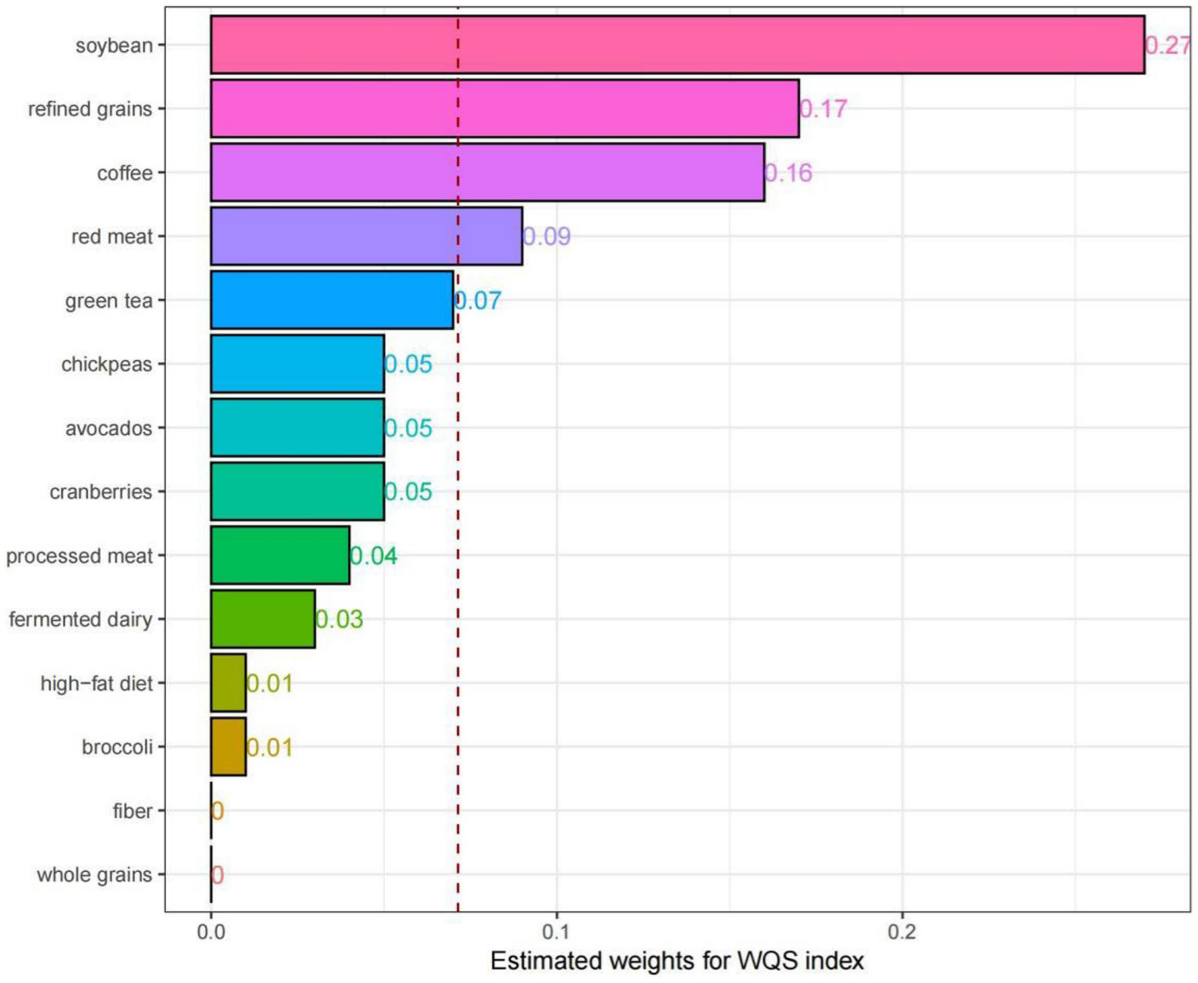
Weighted quantile sum (WQS) model regression index weights for the NAFLD, adjusted for age, sex, race, education attainment, marital status, BMI, PIR, smoking status, hypertension, DM, CVD, ALT and AST.

A weighted index was developed based on the WQS regression to assess the impacts and weight contributions of different DI-GM components on NAFLD (Figure 4). Herein, consistent variable adjustments were applied. The results demonstrated that soybean (27%), refined grains (17%), coffee (16%) and red meat (9%) had high weight contributions, while fiber (0%) and whole grains (0%) had low weight contributions.

## Discussion

As demonstrated, DI-GM remained an independent risk factor for NAFLD after variable control. According to the RCS curves, DI-GM had negligible non-linear, dose-dependent association with NAFLD. Additionally, DI-GM was negatively related to the probability of NAFLD, suggesting that DI-GM can serve as an effective indicator for NAFLD, and adjustment of the diet structure could aid in preventing NAFLD.

In previous studies, DI-GM has been found to be associated with biological age through the mediation role of body mass index, while biological age is linked with various biomarkers possibly in relation with NAFLD (29). After consulting relevant literature, gut microbiota can interact with the pathogenesis of NAFLD by various mechanisms: (1) gut microbiota translocation: dysbiosis of gut microbiota induces translocation of bacteria or their metabolites from gut to liver, causing liver inflammation and damage (35–38); (2) production of endogenous ethanol: gut microbiota can produce endogenous ethanol, which may affect the metabolism and inflammatory response of liver (36, 39–44); (3) abnormal regulation of bile acid and choline metabolism: gut microbiota participates in the metabolism of bile acids and choline, and changes in these metabolites may affect liver health (22, 36, 41, 45–48); (4) endotoxemia: dysbiosis of gut microbiota could result in increased level of endotoxins (e.g., lipopolysaccharides), resulting in inflammation and insulin resistance (36, 49–53). Overall, GM is closely related to NAFLD, with a two-way influence through the gut-liver axis regulatory mechanism (22, 35, 36, 46, 49, 54–57). Therefore, a balanced gut microbiota helps maintain hepatic homeostasis, while disruption of GM may promote the incidence and progression of NAFLD.

The associations of various dietary indicators and the NAFLD have been thoroughly investigated. It has been demonstrated that specific dietary patterns could significantly reduce the risk of MASLD and relevant liver fibrosis (MASLD-LF) (58). Meanwhile, high scores of the HEI or the AHEI were significantly correlated with low incidence of MAFLD (59), and high-quality diets led to low mortality rates of MAFLD (60). To date, several dietary assessment tools, including HEI and AHEI, have been employed to evaluate dietary quality. Nevertheless, they barely considered the specific correlations of dietary components with gut microbiota, which are essentially important for liver health (61).

The DI-GM is distinctive in that it specifically examines the impact of the gut microbiota, addressing a gap in existing dietary indices. Based on the integrated effects of 14 key dietary components on gut microbiota, the DI-GM’s structured approach not only offers insights into dietary impacts on the microbiota but also reflects overall dietary quality (26). The DI-GM provides an assessment framework, making it exceedingly valuable for exploration of the relationship between microbiota and metabolic health. Its correlation with the HEI exemplifies the dual applicability and scientific relevance of the DI-GM in assessing nutritional status and gut microbiota (26). Therefore, future dietary interventions could leverage the DI-GM to optimize dietary structures, thereby improving the conditions of GM and relieving NAFLD.

The results of the WQS regression model indicate that soy, refined grains, coffee, and red meat are key food components influencing NAFLD. Among them, soybean and coffee, as beneficial components of the DI-GM, may play critical roles in the prevention and management of NAFLD. Indeed, soybean can regulate lipid metabolism and oxidative stress, thus protecting the liver of NAFLD patients (62). Consequently, soybean may serve as an effective dietary intervention for NAFLD (62). Some studies claimed that coffee consumption could reduce the risk of NAFLD (63), while conflicting results regarding its impact on prevention of NAFLD have also been reported. For NAFLD patients, coffee intake can relieve liver fibrosis (64). On the contrary, refined grains and red meat, as detrimental components of the DI-GM, may elevate the risk of NAFLD. Indeed, the consumption of refined grains led to increased incidence of NAFLD, while the intake of whole grains may positively affect the clinical conditions of NAFLD patients and relieve disease progression (65). Furthermore, the consumption of red meat has been confirmed to have a positive correlation with NAFLD risk (66). Eating one serving of legumes per week instead of red or processed meats or poultry led to reduced incidence of NAFLD (67). However, the mechanism by which these foods influence the pathogenesis of NAFLD by modulating gut microbiota remains unclear. Moreover, the content of GM in dietary fiber and whole grains were relatively low, which may lead to the 0% of dietary fiber and whole grains of the DI-GM in the contributions of NAFLD.

This study provides epidemiological evidence supporting the inverse association between DI-GM scores and NAFLD prevalence. These findings underscore the potential utility of DI-GM optimization as a preventive strategy in metabolic health management. To advance clinical translation, future longitudinal investigations should employ standardized DI-GM assessments across diverse cohorts to establish causal relationships, validate risk prediction models, and facilitate early risk stratification in susceptible populations.

### Contributions and limitations

This study made contributions in several aspects. First, a broad cross-sectional approach leveraging NHANES data was used for the first time, revealing a linear negative correlation of the DI-GM related to GM diversity and NAFLD. Second, sampling weights, variable adjustment, and statistical tools were involved, resulting in significantly improved precision and robustness. Third, a WQS regression model was employed to evaluate the overall impacts and their weights of different DI-GM components on NAFLD.

Nevertheless, this study has several limitations: (1) the causality of DI-GM and NAFLD cannot be determined due to the cross-sectional approach used; (2) the findings may not be applicable for other populations; (3) recall bias arises from self-reported 24-h dietary records, which weakens the reliability of our results; (4) the confounding effects induced by measurement errors in unknown confounding factors or unmeasured variables cannot be completely ruled out.

## Conclusion

DI-GM can effectively assess the influences of diet on GM, and it is negatively correlated with NAFLD risk. Soybean, refined grains, coffee, and red meat are key food components influencing NAFLD. Future studies may focus on the direct correlation of DI-GM with NAFLD, assessment of the effectiveness of prevention and treatment of NAFLD by improving DI-GM scores through dietary interventions.

## Data Availability

Publicly available datasets were analyzed in this study. This data can be found: the official website of the National Center for Health Statistics (URL: https://www.cdc.gov/nchs/nhanes).
