# Supplementary material for: A study of correlation of the dietary index for gut microbiota with non-alcoholic fatty liver disease based on 2007–2018 National Health and Nutrition Examination Survey
Source: Front Nutr. 2025 Apr 10;12:1573249. doi: 10.3389/fnut.2025.1573249 (PMC12018250; doi:10.3389/fnut.2025.1573249)
Supplement: Supplementary file 1 [file Table_1.docx]

**Table S1. Components and scoring criteria of DI-GM in NHANES.**

| **Components of DI-GM** | **Food items included in NHANES** | **Scoring criteria** |
| --- | --- | --- |
| **Beneficial to gut microbiota** | Avocados | Score 1 - Consumption≥sex-specific median  Score 0 - OtherwiseGreen tea |
|  | Broccoli |  |
|  | Chickpeas |  |
|  | Coffee |  |
|  | Cranberries |  |
|  | Fermented dairy (including yogurt, cheese, kefir, sour cream, buttermilk) |  |
|  | Fiber |  |
|  | Green tea |  |
|  | Soybean (including Soy milk, Tofu) |  |
|  | Whole grains |  |
| **Unfavorable to gut microbiota** | Refined grains | Score 0 - Consumption≥sex-specific median  Score 1 - Otherwise |
|  | Processed meat |  |
|  | Red meat |  |
|  | High-fat diet (% energy) | Score 0 - Consumption≥40%  Score 1 - Otherwise |

Abbreviations: DI-GM, dietary index for gut microbiota; NHANES, National Health and Nutrition Examination Survey.

**Table S2. The detail definition and classification of covariates.**

| **Variables** | **Definitions or Classification** |
| --- | --- |
| Sex | Male, Female. |
| Race | Non-Hispanic White, Non-Hispanic Black, Mexican American, Other Race. |
| Education attainment | High school or less, More than high school. |
| Marital status | Married or living with partner, Living alone. |
| PIR | Low (PIR ≤1), Middle (1< PIR < 4), and High (PIR ≥4). |
| Smoking status | Never smoking: <100 cigarettes in lifetime;  Former smoking: >100 cigarettes in life and smoke not at all now;  Now smoking: >100 cigarettes in lifetime. |
| Hypertension | An average systolic blood pressure (SBP) equal to or exceeding 140 mmHg;  An average diastolic blood pressure (DBP) equal to or exceeding 90 mmHg;  Self-reported hypertension;  Individuals taking prescribed anti-hypertensive medications. |
| DM | i) physician confirmation of diabetes diagnosis, ii) glycohemoglobin levels equal to or greater than 6.5%, iii) fasting glucose ≥ 7.0 mmol/L, iv) random blood glucose≥ 11.1 mmol/L, and v) documented use of DM medication. |
| CVD | The medical conditions section, identified by the variable name prefix MCQ, encompasses self- and proxy-reported personal interview data covering an extensive range of health conditions and medical history for both children and adults. This section includes inquiries such as ‘Has a doctor or other health professional ever informed you/SP that you/he/she… had congestive heart failure, coronary heart disease, angina (also called angina pectoris), heart attack (also called myocardial infarction), stroke, etc.?’ These questions, labeled as MCQ160B-F in the household questionnaires administered during home interviews, were utilized to identify participants with a history of CVD if they responded ‘yes’ to any of these questions. |
| Laboratory tests | The Specific method can be found in this webpage  ([https://wwwn.cdc.gov/nchs/nhanes/continuousnhanes/labmethods.aspx?BeginYear=200](https://wwwn.cdc.gov/nchs/nhanes/continuousnhanes/labmethods.aspx?BeginYear=2017)7) |

Abbreviations: PIR, family poverty income ratio; DM, diabetes mellitus; CVD, cardiovascular disease.

**Table S3. Sensitivity analysis of DI-GM and NAFLD.**

| **Characteristic** | **OR (95%CI), P value** |
| --- | --- |
| DI-GM (continuous) | 0.91 (0.85,0.99), 0.019 |
| DI-GM (categorical) |  |
| 0-3 | Reference |
| 4 | 0.86 (0.61,1.20), 0.361 |
| 5 | 0.88 (0.64,1.23), 0.458 |
| >=6 | 0.66 (0.48,0.91), 0.012 |
| *P* for trend | 0.016 |

Adjusted for age, sex, race, education attainment, marital status, BMI, PIR, smoking status, hypertension, DM, CVD, ALT, AST, physical activity, alcohol intake, and medication use.
